# Genetic Variations Strongly Influence Phenotypic Outcome in the Mouse Retina

**DOI:** 10.1371/journal.pone.0021858

**Published:** 2011-07-14

**Authors:** Austin S. Jelcick, Yang Yuan, Barrett D. Leehy, Lakeisha C. Cox, Alexandra C. Silveira, Fang Qiu, Sarah Schenk, Andrew J. Sachs, Margaux A. Morrison, Arne M. Nystuen, Margaret M. DeAngelis, Neena B. Haider

**Affiliations:** 1 Department of Genetics, Cell Biology and Anatomy, University of Nebraska Medical Center, Omaha, Nebraska, United States of America; 2 Ocular Molecular Genetics Institute, Department of Ophthalmology, Harvard Medical School, Massachusetts Eye and Ear Infirmary, Boston, Massachusetts, United States of America; 3 Division of Biostatistics, University of Nebraska Medical Center, Omaha, Nebraska, United States of America; 4 Moran Eye Center, University of Utah, Salt Lake City, Utah, United States of America; 5 Department of Ophthalmology, Harvard Medical School, Schepens Eye Research Institute, Boston, Massachusetts, United States of America; The National Institute of Diabetes and Digestive and Kidney Diseases, United States of America

## Abstract

Variation in genetic background can significantly influence the phenotypic outcome of both disease and non-disease associated traits. Additionally, differences in temporal and strain specific gene expression can also contribute to phenotypes in the mammalian retina. This is the first report of microarray based cross-strain analysis of gene expression in the retina investigating genetic background effects. Microarray analyses were performed on retinas from the following mouse strains: C57BL6/J, AKR/J, CAST/EiJ, and NOD.NON-*H2* ^-nb1^ at embryonic day 18.5 (E18.5) and postnatal day 30.5 (P30.5). Over 3000 differentially expressed genes were identified between strains and developmental stages. Differential gene expression was confirmed by qRT-PCR, Western blot, and immunohistochemistry. Three major gene networks were identified that function to regulate retinal or photoreceptor development, visual perception, cellular transport, and signal transduction. Many of the genes in these networks are implicated in retinal diseases such as bradyopsia, night-blindness, and cone-rod dystrophy. Our analysis revealed strain specific variations in cone photoreceptor cell patterning and retinal function. This study highlights the substantial impact of genetic background on both development and function of the retina and the level of gene expression differences tolerated for normal retinal function. These strain specific genetic variations may also be present in other tissues. In addition, this study will provide valuable insight for the development of more accurate models for human retinal diseases.

## Introduction

The amount of genetic diversity within a species is significant and contributes to the survival of the species through genetic drift. There are many examples of sequence variants which have no detrimental functional consequences. These variants result in a range of normal phenotypes as well as those associated with disease. For example, iris pigmentation is an easily observable trait that varies with genetic background and has no detrimental consequence [1]. Other observable phenotypic changes are non-disease associated, and result in the biological diversity seen between individuals. These can include alterations in skin pigmentation, hair pigmentation, height, and other non-disease associated traits.

Genetic variation can also have a negative impact. Disease associated phenotypic differences such as severity and progression rate can be the result of single gene mutations and different mutations within the same gene causing unique phenotypes [2]. In addition, quantitative trait loci (QTL) and modifier genes are examples of mechanisms where multiple sequence variants work in concert to produce a single phenotype [3]. Thus, variations in disease phenotype can vary not only between individuals, but also within a given gene. There are many examples in humans of variation in disease phenotype due to variation in genetic background, including rheumatoid arthritis, coeliac disease, and interstitial lung disease [4]–[5]. Genetic disorders can often exhibit large variations in disease expressivity and penetrance that can be attributed to allelic differences or genetic background effects. The influence of genetic background on disease phenotype is commonly observed in mice [6], [7]–[11]. However, the underlying genetic determinants responsible for many phenotypic variances are still poorly understood.

There are over 200 inbred mouse strains, each with unique genetic and phenotypic traits (http://jaxmice.jax.org/research/index.html). Phenotypic variations in inbred mouse strains have served as models for many human diseases, and are used in the development of new therapies. Such diseases include obesity [12], seizure threshold [13], alcohol consumption [14], visual acuity [15], complete agenesis of the corpus collosum [16], and aggressive behavior [17].

Genetic alterations have a significant impact on the retina. Over two-thirds of retinal diseases result in degeneration of rod and/or cone photoreceptor cells, which comprise 70% of all retinal cells. Phenotypic variations in the retina include disease onset and severity, such as that observed for the retinal degeneration 7 (*rd7*) mouse model, which harbors a mutation in the nuclear hormone receptor *Nr2e3* [18] and models the human retinal disease enchanced S-cone syndrome. The identification of underlying genetic determinants and their associated pathways is thus vital to further understanding the influence of genetic variation on phenotypic variation and severity.

The inbred strains used in this study are homogenous within the strain yet highly genetically divergent from one another [9]. Three of the strains (CAST/EiJ, AKR/J, NOD.NON-*H2* ^-nb1^) are known to harbor suppressor alleles for retinal degeneration associated with the *rd7* phenotype [6] while one strain, C57BL6/J (B6), exhibits 100% penetrance for the disease. These strains have also been shown to harbor modifier alleles for other phenotypes. For example, the AKR/J mouse harbors modifier alleles for *RS1* related X-Linked retinoschisis [19] and TUB related retinal degeneration [20]. Similarly, the CAST/EiJ mouse harbors modifier alleles for *EYA1* related cochlear aplasia [21], Apc (Min) related intestinal polyps [22], and can develop coloboma. Additionally, micro- or anophthalmia is frequently observed in C57BL6/J mice (our unpublished observations). These strains are derived from both wild and inbred mice. The diverse genetic backgrounds of the strains used, and the inclusion of strains not noted for limited polymorphism ensures a more accurate analysis of differential expression between strains [23].

The purpose of this study was to determine the impact of differential gene expression, on the morphology and function of the developing and mature mouse retina. The study was performed using four genetically divergent inbred strains of mice (C57BL6/J; AKR/J; CAST/EiJ; NOD.NON*-H2* ^-nb1^) that were previously shown to harbor modifier alleles for retinal degeneration [18]. Each strain has a unique signature gene expression that impacts whole gene networks temporally. These signature strain specific differences translate into biological consequences affecting cell patterning and retinal function. This study thus provides valuable insight into the consequence of varying gene expression on biological processes. These data will further aid in the development of more appropriate mouse model systems that recapitulate human disease, which may ultimately impact disease causality and response to therapies.

## Results

### Microarray Expression Analysis and Confirmation

**Figure 1:**
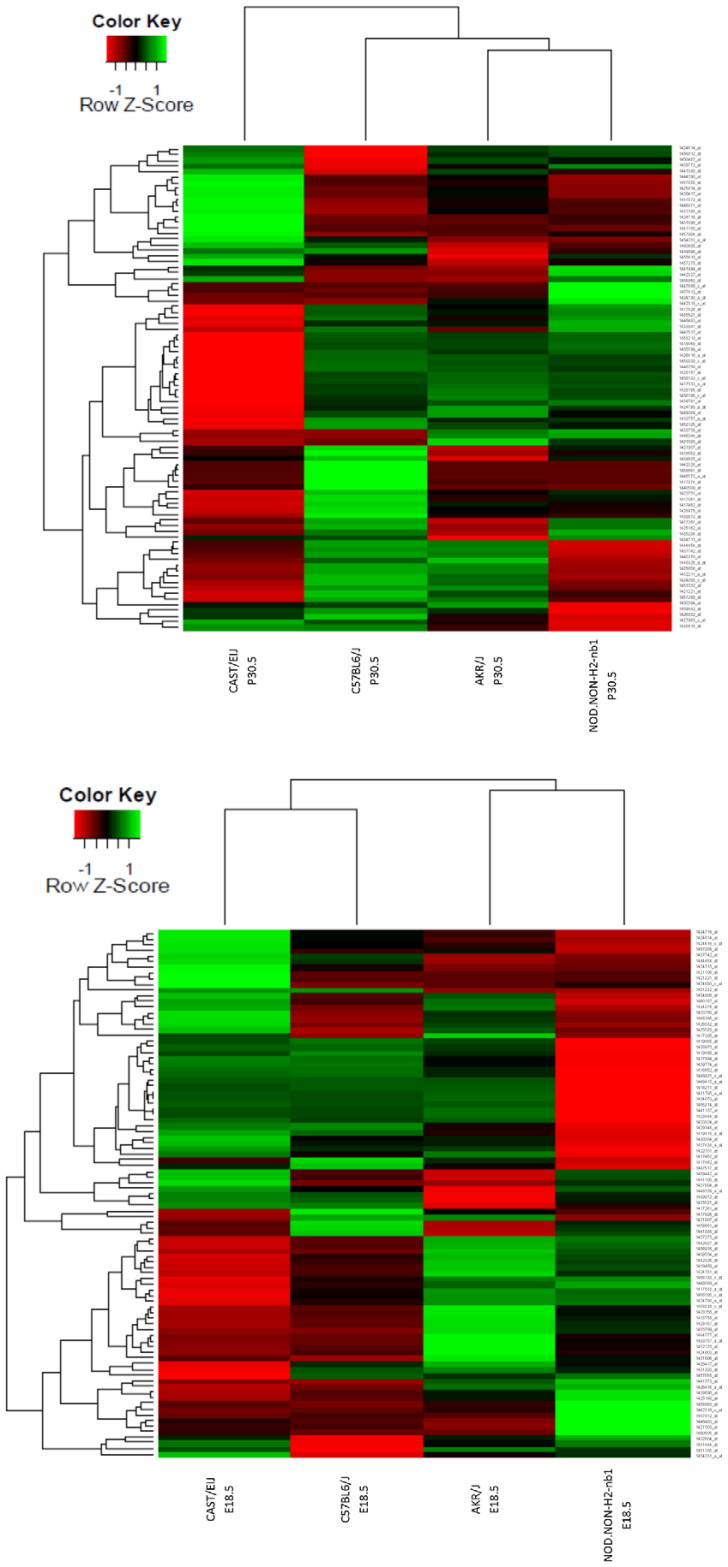
Heatmap of Gene Expression in CAST/EiJ; C57BL6/J; AKR/J; and NOD.NON-H2-nb1. Color key represents the relative expression for a gene for each strain based on the estimated mean log2 intensities. Rows represent the individual genes, while columns represent each strain. Top heat map depicts expression and average-linkage hierarchical clustering for differentially expressed genes at P30.5 from each pair comparison relative to B6. Bottom heat map depicts expression and average-linkage hierarchical clustering for differentially expressed genes at E18.5 from each pair comparison relative to B6.

To determine the impact of genetic background on differential gene expression, we performed microarray analysis on retinas from C57BL6/J, CAST/EiJ, AKR/J, and NOD.NON*-H2* ^-nb1^ mice at E18.5 and P30.5. Expression variances were identified by pair-wise comparisons of each strain relative to C57BL6/J utilizing criteria of a 2 fold change or greater and a p-value <0.001 adjusted for a false discovery rate of 10% (BRB Arraytools). Significant strain specific variations in expression were identified at both E18.5 and P30.5, clearly visible by heatmap (Figure 1). Expression data was confirmed by qRT-PCR, with over 82% of the 138 genes tested confirmed. Genes involved in retinal function, such as the perception of external stimuli, retinoic acid receptor activity, axon guidance, photoreception, and neuronal development had a greater than 90% confirmation rate (Table 1). We also found 3098 and 2685 differentially expressed genes to be highly statistically significant at E18.5 and P30.5 respectively (Table S1). A subset of the statistically significant genes (120) from the E18.5 dataset were selected for further analysis based on the following criteria in addition to their initial statistical significance: 1) a known role in retinal development; 2) an association with retinal disease; 3) a wide disparity in inter-strain expression. Ontological analysis was performed on these genes and generated three major networks (Table 1). Additionally, we observed temporal variance in gene expression across all strains.

**Table 1:** Confirmation of Gene Expression via qRT-PCR.

	Gene	Strain	Timepoint	Microarray Fold Change	qRT-PCR Fold Change
**Transcription/Signal Transduction Network**					
**(Network 1)**	Foxc1	AKR/J	E18.5	10.02	17.45
	Rarg	AKR/J	E18.5	2.61	12.00
	Gng2	CAST/EIJ	E18.5	−11.11	−1.31
	Ncoa2	CAST/EIJ	E18.5	1.29	1.69
	Nrip1	CAST/EIJ	E18.5	−2.78	−1.69
	Rarb	CAST/EIJ	E18.5	2.56	1.71
	Rbl1	CAST/EIJ	E18.5	−5.00	−1.86
	Sfrp1	CAST/EIJ	E18.5	3.69	3.65
	Cd151	CAST/EIJ	P30.5	2.74	−1.19
	Itgb5	CAST/EIJ	P30.5	−11.11	−54.33
	Kif4	CAST/EIJ	P30.5	−1.18	−3.09
	Med1	CAST/EIJ	P30.5	−4.17	−2.87
	Nfatc2	CAST/EIJ	P30.5	2.07	1.53
	Nr2c1	CAST/EIJ	P30.5	−2.38	−3.06
	Nr2c2	CAST/EIJ	P30.5	−1.72	−1.70
	Opn1sw	CAST/EIJ	P30.5	−1.67	1.14
	Prrx1	CAST/EIJ	P30.5	−1.39	−2.05
	Thrap3	CAST/EIJ	P30.5	−12.50	−3.23
	Tle4	CAST/EIJ	P30.5	3.20	−1.59
	Nsd1	NOD.NON-H2-nb1	E18.5	−1.89	−1.91
	Sema3b	NOD.NON-H2-nb1	E18.5	1.25	1.03
**Photoreception/Development Network**					
**(Network 2)**	Nisch	CAST/EIJ	E18.5	−16.67	−4.37
	Rai14	CAST/EIJ	E18.5	−5.26	−2.94
	Retsat	CAST/EIJ	E18.5	6.18	1.17
	Rpgrip1	CAST/EIJ	E18.5	−4.00	−4.73
	Trp53bp1	CAST/EIJ	E18.5	−20.00	−1.63
	Crabp1	CAST/EIJ	P30.5	2.45	1.48
	Rab21	CAST/EIJ	P30.5	2.51	−1.28
	Rab37	CAST/EIJ	P30.5	−1.11	−1.25
	Rgs9	CAST/EIJ	P30.5	−10.00	−1.79
	Scmh1	CAST/EIJ	P30.5	−2.08	−4.33
	Tle1	CAST/EIJ	P30.5	2.13	−1.24
	Vsx1	CAST/EIJ	P30.5	−1.10	−25.43
	Mitf	NOD.NON-H2-nb1	E18.5	2.84	4.51
**Cell Communication Network**					
**(Network 3)**	Crxos1	AKR/J	E18.5	−2.00	−10.98
	Jag1	CAST/EIJ	E18.5	3.03	2.03
	Abcd2	CAST/EIJ	P30.5	−1.16	−23.11
	Fbxo2	CAST/EIJ	P30.5	1.21	−2.71
	Fbxo9	CAST/EIJ	P30.5	1.98	−1.27
	Rorb	CAST/EIJ	P30.5	1.34	−2.53
	Spata5l1	CAST/EIJ	P30.5	−3.85	−1.38
	Zfp365	CAST/EIJ	P30.5	2.55	1.47
	Sema3c	NOD.NON-H2-nb1	E18.5	4.32	5.81
	Rbp3	NOD.NON-H2-nb1	E18.5	−3.03	−3.91
	Sox30	NOD.NON-H2-nb1	P30.5	10.57	65.83

Genes found statistically significant by microarray that were tested via qRT-PCR. Fold changes found by microarray and qRT-PCR are shown, as well as strain and time point in which the gene was tested.

**Figure 2:**
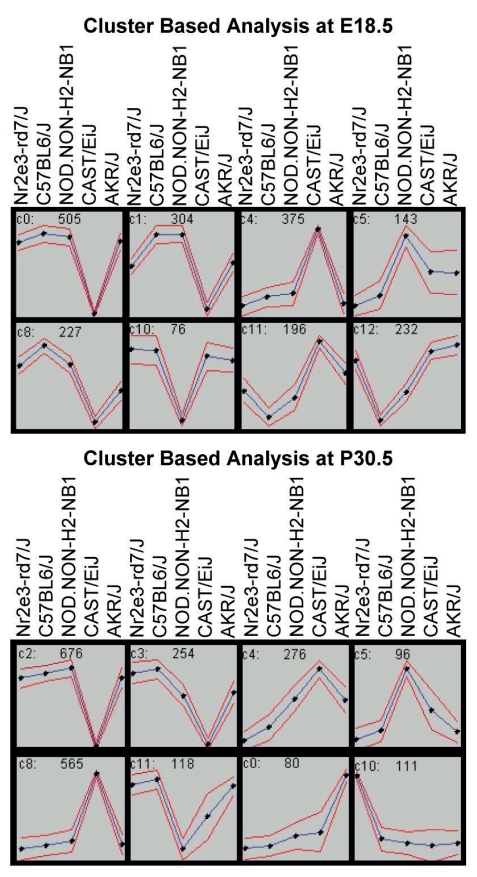
Cluster Based Analysis of Strain Specific and Temporal Gene Expression. Cluster based analysis showing significant strain specific gene expression at E18.5 and P30.5. X axis designates strain/time while the Y axis depicts standardized mean log2 intensity. The number in each cluster graph is the number of genes within each cluster.

To further determine strain specific and temporal gene expression, clustering analysis using GeneCluster 2 was performed. Multiple clusters containing highly significant genes were generated. These clusters were generated based on 1) statistical significance of differential expression 2) expression unique to a given strain (i.e. up-regulated in AKR/J); these clusters verified our prior observation of strain specific and temporal gene expression. These clusters were analyzed to determine their involved pathways. AKR/J specific genes functioned in DNA binding and transcription, cell cycle and phosphorylation; CAST/EiJ specific genes functioned in apoptosis, protein localization, synaptic transmission and cell morphogenesis; while genes specific to E18.5 functioned cell cycle, phosphorylation, neurological function, and apoptosis. A subset of these clusters featuring strong strain specific expression at both E18.5 and P30.5 is shown (Figure 2). These results further illustrate the impact of genetic background on gene expression as well as temporal gene expression during development.

### Determination of Gene Ontologies and Functional Relationships of Differentially Expressed Genes

Pathway analysis was performed to identify key gene networks that were differentially expressed. Analysis of these differentially expressed genes revealed conserved top scoring networks, network functions, and canonical pathways relative to C57BL6/J. Specific genes exhibiting the greatest variation differed between strains and network involvement (Table S2). At P30.5, three major networks were identified functioning in neurological disease, nervous system development and function, and axonal guidance; while at E18.5 three networks were identified functioning in cell signaling, cellular assembly and cellular organization. Interestingly, CAST/EiJ showed the greatest expression variation (as great as 147 fold) between strains, such as with its down regulation of Nyctalopin (NYX) (−4.42 fold, p<7.92x10^−8^).

Pathway analysis of the 120 gene subset was performed to determine additional gene function. This analysis revealed involvement in transcription, signal transduction, sensory perception, and cellular communication (Network 1); photoreception, development, and metabolism (Network 2); and cell communication (Network 3). The genes within these networks varied in individual function, but showed primary involvement in transcription (41%), visual perception (13%), and signal transduction/cell communication (6%). Each network showed strain specific variation, while Networks 1 and 2 also showed temporal variation. These networks show more specialized function in the retina relative to the initial three networks. However, variation within both networks suggests phenotypic consequences resulting from expression variances are not limited to the retina.

Interestingly, genes showing both strain specific and temporal variation include genes which have been implicated in retinoblastoma (*RBL1*; [24]), focal retinal ganglion cell loss (*THRAP3*; [25]), and retinal development (*NCOA2*; [26], *CRABP1* [27], *RGS9* [28], and *TLE1* [29]). *RGS9* for example, is associated with Bradyopsia in humans [30], which is characterized by significant down regulation in P30.5 CAST/EiJ (10.1 fold, p<2.27×^10−8^), and decreased electroretinogram amplitudes, correlates or mimics the response suppression observed in affected humans.

### Analysis of Functional Gene Networks

**Figure 3:**
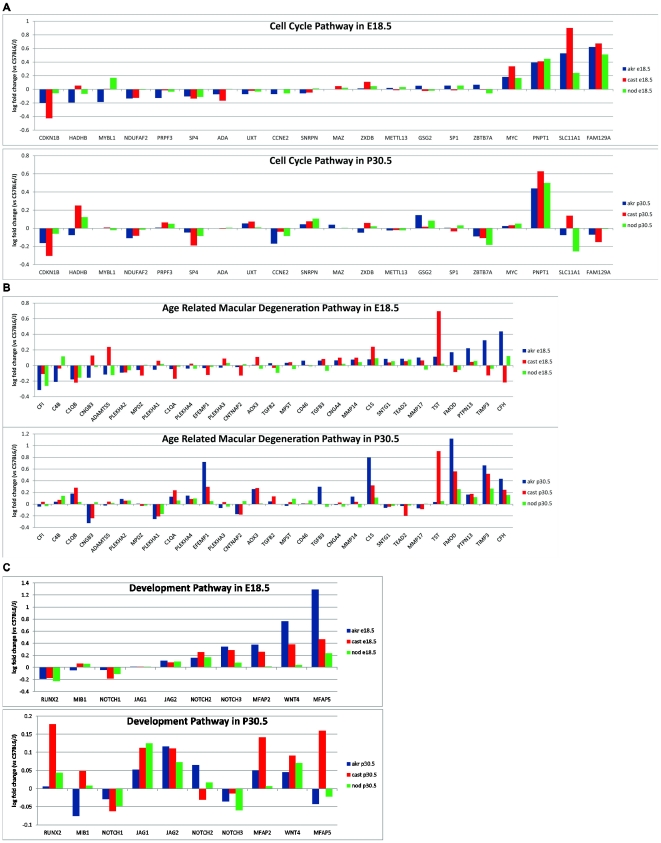
Key Pathway Analyses of Cell Cycle; AMD; and Development. Gene expression levels for genes present in the cell cycle, age related macular degeneration, and development pathways at E18.5 and P30.5 are shown. Expression levels for each strain are relative to C57BL6/J.

Analysis of networks based on genes involved in cell cycle, age related macular degeneration (AMD), and development were also performed showing temporal and strain specific variation in expression. Rationale for the retinal disease pathway was based on findings that genetic alterations specific to CAST/EiJ have been shown to be potentially involved in the development of age related macular degeneration (AMD) [31]. The AMD network was generated utilizing several AMD associated genes [32]–[52]. The cell cycle, AMD, and development pathways exhibited strain specific variation in expression (Figure 3).

A developmental network (Figure 3) was also generated based on *NOTCH*, *WNT*, and *JAG* which are known to be involved in brain development [53], inner ear development [54], response to Vitamin D [55], and retinal development [56], [57]. This network showed strain specific variation in expression as well as temporal variances. These temporal expression variances are further supported by current studies showing *NOTCH* and *WNT* temporal expression is involved in retinal development [56], [57].

### Functional Correlation of Gene Expression with Observed Phenotypes

**Figure 4:**
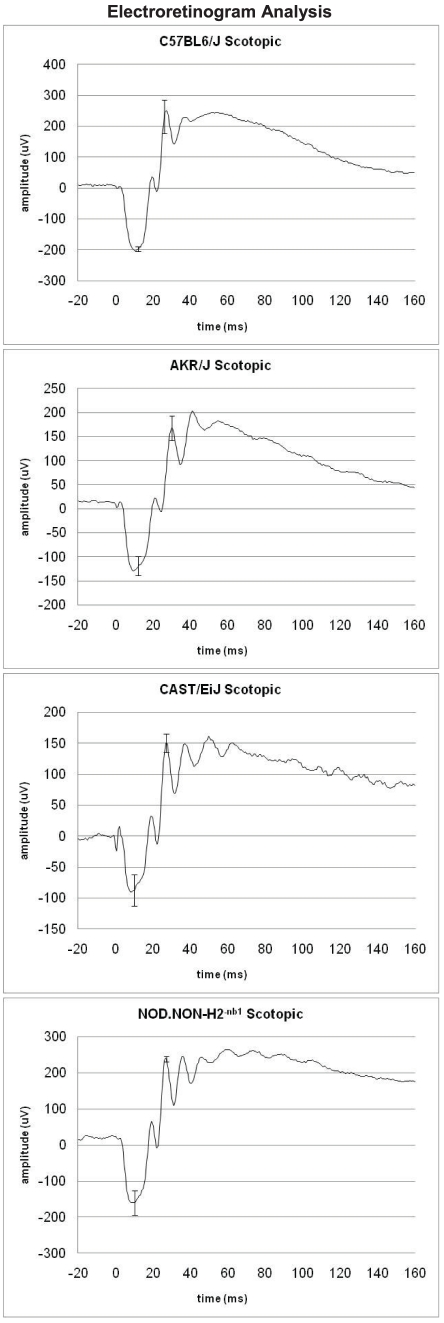
Scotopic Electroretinogram Analysis. ERG analysis is shown for each strain under scotopic conditions. Each strain represents the average of readings for three animals. Amplitude is measured in micro-volts (u) while time is measures in milliseconds (ms) with error bars shown for the A and B-waves respectively.

Electroretinograms were performed to determine if genetic background influences retinal function (Figure 4). Rod and cone photoreceptor cell function are depicted by the amplitude and temporal length of the a-wave and communication to second order neurons by the b-wave. Each strain exhibited unique retinal responses to light stimuli. Our previous report focused on cone photoreceptor (photopic, light adapted) function and showed CAST/EiJ retinas have reduced cone response compared to NOD.NON*-H2* ^-nb1^ and C57BL6/J [9]. In this study, we examine rod photoreceptor (scotopic, dark adapted) function. Overall, C57BL6/J retinas showed the greatest amplitudes of scotopic a- and b-waves while AKR/J retinas exhibit the smallest scotopic b-wave response (Figure 4). These strain specific variations in retinal response mimic human retinal disease. This similarity adds support for these strains as models for human disease.

### Variation in Genetic Background Influences Protein Expression in the Retina

**Figure 5:**
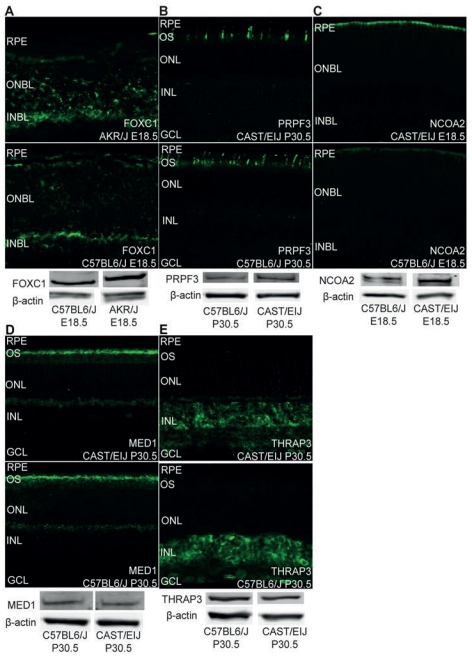
Western Blot and Immunohistochemical Analysis of Protein Expression and Localization. Western blots and immunohistochemical staining of retinal cross sections using antibodies FOXC1, PRPF3, NCOA2, MED1, and THRAP3. Each was found to be differentially expressed at the transcript level by microarray and qRT-PCR. Western Blot expression levels are in reference to levels of beta-actin. Cross sections used for immunohistochemistry are matched for orientation and field of view. Cross sections of E18.5 retinas are labeled with the following: retinal pigment epithelium (RPE), outer neuroblastic layer (ONBL), and inner neuroblastic layer (INBL). Cross sections of P30.5 retinas are labeled with the following: retinal pigment epithelium (RPE), outer segment (OS), outer nuclear layer (ONL), inner nuclear layer (INL), and ganglion cell layer (GCL).

Western blot and immunohistochemical analysis was performed on five confirmed differentially expressed genes to determine if differences in mRNA expression impact protein expression and (Figure 5). These genes were selected based on their involvement in our first top scoring gene network. This network features genes present in the *TRβ* pathway. Pathway analysis of *TRβ* shows interaction with *OPN1SW*. Genes with tested antibodies were used for western blotting and immunohistochemistry. The majority of genes examined exhibited differential protein expression consistent with differential transcript expression. Localization of *PRPF3*, *NCOA2*, *MED1*, and *THRAP3* protein was consistent between strains while localization of *FOXC1* varied.

### Strain Specific Variations of Cone Cell Topography, *OPN1SW* Distribution, and Cone Specific Genes

**Figure 6:**
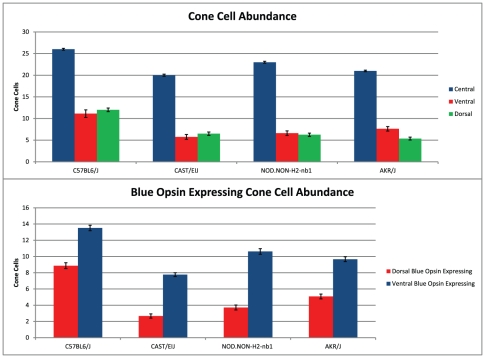
Cone Cell and OPN1SW Expressing Cone Cell Abundance across Strains. Cone cell counts within the central retina, dorsal and ventral retina, and those for dorsal and ventral cone cells expressing blue opsin are shown for each strain.

**Figure 7:**
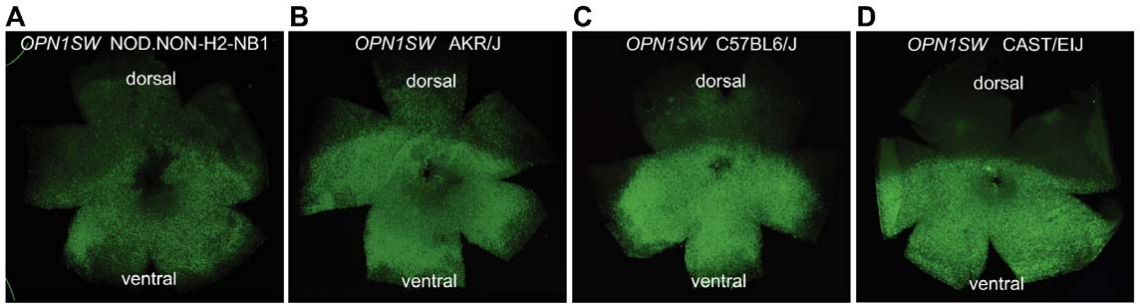
Retinal Whole Mounts Labeling OPN1SW for NOD.NON-H2-nb1; AKR/J; C57BL6/J; and CAST/EiJ. Immunohistochemical analysis of retinal whole mounts for each strain labeling blue opsin (OPN1SW). Whole mounts are orientation matched, with dorsal and ventral being labeled.

Blue opsin expression occurs in a dorsal-ventral gradient distribution in the mouse retina [58], with approximately one third of the retina showing little or no blue opsin expression. To determine if retinal topography varies between strains, we examined whole mounts of P30.5 retinas from each strain labeled with peanut lechtin (PNA) to label all cones, and blue opsin (*OPN1SW*) to label blue cones. Total cone and blue opsin expressing cone cell abundance was measured within central, dorsal, and ventral retina in a 500 um region (Figure 6; Figure 7).

Total cell quantification showed C57BL6/J to have the greatest abundance of cone cells. Between the other strains, CAST/EiJ exhibited the greatest number of dorsal cone photoreceptor cells, the fewest ventral, and the fewest cone photoreceptor cells overall. Similarly, AKR/J had the fewest dorsal cone photoreceptor cells, but the greatest ventral. Additionally, NOD.NON*-H2* ^-nb1^ had the greatest overall cone cell abundance. For blue opsin expressing cone cells, C57BL6/J also showed the greatest abundance. Between other strains, CAST/EiJ retinas showed the fewest number in both the dorsal and ventral retina, AKR/J the greatest dorsal number, and NOD.NON*-H2* ^-nb1^ the greatest ventral number. This critical finding suggests that high abundance of cone cells does not imply a high expression of blue opsin. This confirms our previous finding that total cone cell abundance does not imply high abundance within a specific retinal region. Interestingly the expression levels of several cone cell specific genes [59] correlate with total and blue opsin expressing cone cell abundance. C57BL6/J showed the highest expression of all cone specific genes (Figure S1).

## Discussion

Genetic background can have a profound impact on both disease and non-disease associated phenotypes. We determined that genetic background impacts whole gene networks, key developmental processes that establish normal retinal topography, and ultimately influences phenotypic outcome associated with human retinal disease. These findings have wide reaching impacts as they each substantiate the fact that genetic background can not only influence disease phenotypes, but that non-diseased, “normal” phenotypes can show variations as a function of genetic background as well. Signature expression profiles identified three major biological networks and several tangent networks. The influence of genetic background on the expression of these networks shows strain and temporal specific variation. Putative network function analyses suggest that genetic background influences biological processes affecting cell signaling and cellular organization.

In this study, we determined a key correlation between retinal topography and genetic background. In the mammalian retina, cone photoreceptor cells are distributed in a defined pattern across the retina. In humans and non-human primates, there is a macula near the center of the eye. Within the macula is the fovea, a cone dense region comprised solely of red and green cones. This region of the eye provides the highest point of visual acuity. Genetic defects affecting the macula include cone dystrophies and macular degeneration, and affect the central vision. Humans and non-human primates possess a single fovea while bird species such as hawks are bifoveal, and dogs and cats lack a fovea but have a central band known as a visual streak (http://www.diabetesdaily.com/wiki/Retina). The mouse retina does not have a macula or fovea proper, however there is gradient in expression of the opsin genes in mouse cone cells. Green opsin has a uniform pattern of expression while blue opsin is expressed in a dorsal to ventral gradient, with a ventral concentration [58]. In this study, we determined that while the general pattern of cone photoreceptor cells and blue opsin expression is conserved, there are pronounced differences in patterning between mouse strains. Using these “normal” inbred strains, our findings illustrate that genetic background has profound effects on normal cell patterning in the retina. This observation is seen in other eye phenotypes in humans, such as variations in iris pigmentation, corneal thickness, and intra-ocular pressure. These studies thus illustrate the tolerance of the system to impact normal retinal morphology.

The observed changes in retinal function between strains and their correlation with gene expression show that strain specific variations may increase the susceptibility to disease for that strain. Additionally, the variations in expression between strains that show no association with disease illustrate the principle that normal variation between strains exist which are not associated with disease phenotypes. These variations between strains allow their use as models for human ocular diseases while further modeling normal variation between individuals. For example, CAST/EiJ mice exhibit decreased rod function correlating with significantly diminished expression of *NYCTALOPIN* (*NYX*) which models human retinal disease as human mutations in *NYCTALOPIN* are associated with phenotypes associated with impaired rod function, decreased a-wave amplitude, X-linked retinoschisis, and X-linked congenital stationary night blindness [60]-[62]. CAST/EiJ also shows differential expression of *RGS9*; *FOXC1*; *RPGRIP1*; *VSX1*; *RARB*; and *NSD1*. Mutations within these genes have been associated with Bradyopsia; iris hypoplasia with glaucoma [63]; cone-rod dystrophy 13 [64]; corneal dystrophy [65]; Waardenburg syndrome [66]; and Sotos syndrome [67] respectively. We observed significant down-regulation of *RBP3* in NOD.NON*-H2* ^-nb1^ (-7.9 fold, p<3.2×10^−9^), a gene in which mutations have been associated with autosomal recessive retinitis pigmentosa [68]. We further observed two known retinal genes [69], [70] *ROBO2* and *TRPM3*, to be significantly down regulated in CAST/EiJ and NOD.NON*-H2* ^-nb1^ respectively; while also identifying multiple novel genes within our pathways that may be associated with the observed changes in retinal function.

Taken together, the genomic, structural, and functional correlations illustrate the variability tolerated and the level of what is considered “normal”. These studies demonstrate the strong influence of genetic background on phenotypic outcome. The inbred mouse strains, while providing a uniform genetic background are present with unique signature gene expression profiles. These unique gene network/pathway profiles must be taken into consideration when using these to generate targeted genetically engineered animals. Further, by identifying key pathways associated with disease, while accurately noting benign genetic variants, a more targeted approach to understanding disease pathology and future targeted gene therapy design can be achieved.

## Methods

### Ethics Statement

All animals were bred and maintained under standard conditions at The University of Nebraska Medical Center research vivarium in accordance with protocol #04086, approved by the Animal Care and Use Committee at the University of Nebraska Medical Center. Mice were housed in microisolator cages and provided food and water *ad libitum*. The University of Nebraska Medical Center is in compliance with the NIH policy on the use of animals in research (Animal Welfare Act P.L. 89–544, as amended by P.L. 91–579 and P.L. 94–279) as well as the Guide for the Care and Use of Laboratory Animals, NIH Publication No. 86–23.

Mice used in this study were bred and maintained under standard conditions in the research vivarium at the University of Nebraska Medical Center. Tissues were harvested from four genetically divergent strains of mice: B6 (C57 derived strain), CAST/EiJ (wild derived strain), AKR/J (derived from Castle's mice), and NOD.NON*-H2* ^-nb1^ (derived from Swiss mice) [71]. Retinal tissue was collected at embryonic day 18 (E18.5) and postnatal day 30 (P30.5). Adult mice were examined clinically by indirect ophthalmoscopy to examine the fundus.

### Public Access of Microarray Data

All microarray data from this study has been made MIAME complaint and is publicly available through the Gene Expression Omnibus (GEO) database under the series record GSE24512.

### Microarray Analysis

RNA was isolated at E18.5 and P30.5 as previously described [72]. Briefly, eyes were enucleated and placed in PBS on ice. Retinas were dissected using a stereo microscope (Zeiss Stemi SV 11) and RNA was isolated by TRIzol^®^ extraction. A total of 30 retinas were collected from 15 mice of each strain at similar time points during the day. Equimolar amounts of RNA isolated from ten retinas were pooled into three separate pools from each strain and time point. RNA was hybridized to Mouse 420A 2.0 (Affymetrix, Santa Clara, CA) chips by the UNMC Microarray Core Facility according to manufacturer specifications (Affymetrix, Santa Clara, CA). Data quality was assessed using the affyPLM package for the R programming language. Consistency of expression levels was confirmed by validation across multiple redundant probe sets. Differential expression analysis was performed using the Linear Models for Microarray Analysis portion of Bioconductor. Genes found to be differentially expressed for each pair wise comparison using a FDR-adjusted p-value of 0.001 and at least a 2 fold change were combined and used to perform clustering analysis. A self-organizing map (SOM) clustering algorithm was applied to genes showing significant expression differences as judged by mean log2 intensity per strain. The gap statistic was used to estimate the optimal number of clusters. Additional analysis was performed using BRB Array Tools for Excel 2007, as well as the Stanford Statistical Analysis of Microarrays (SAM) plug-in for Excel 2007. Subsequent pathway analysis based on genes found to be statistically significant by these methods was performed using Ingenuity Pathway Analysis software. Gene ontology and further annotation of genes was performed using the Affymetrix NetAffx database, BRB Array Tools, the Database for Annotation, Visualization, and Integrated Discovery (DAVID) (http://david.abcc.ncifcrf.gov), and the UCSC Genome Browser. Specific fold changes for each gene within each network, including fold changes across multiple probes is available for all strains and time points for both the 3098 gene derived networks (Table S3) and the 120 gene derived networks (Table S4).

### Quantitative RT-PCR

Real-time qRT-PCR was performed as previously described [72] to confirm differential expression observed in the microarray data. Greater than 82% of significant gene expression differences were confirmed by quantitative real time-PCR (qRT-PCR) with 45 of the 138 genes tested belonging to top scoring networks. RNA was isolated using the pooled samples from the microarray analysis as well as additional individual samples from E18.5 and P30.5 mice for each strain. First strand synthesis was performed using the RETROscript (Ambion, Austin, TX) on 2 µg of RNA template. Primers were selected using Primer3 software (Table S5). RNA and primers were diluted to 1∶100 concentrations prior to amplification, with qRT-PCR performed utilizing SYBR green PCR master mix (Applied Biosystems, Foster City, CA). qRT-PCR was performed on an ABI 7500 using default cycling parameters. For each RNA sample, triplicate reactions were performed, averaged, and *Δ*Ct normalized to β-actin. Comparisons between strains were made for fold change estimation with relative expression being calculated using the following formula: 1000/2^*Δ*Ctbactin-*Δ*Cttestgene^. Statistical significance of differential expression was determined by T-test using a p-value of <0.05.

### Immunofluorescence

Histological samples were prepared as previously described [73]. Briefly, animals were euthanized and eyes were oriented dorsal to ventral with a cautery, and subsequently fixed in 4% paraformaldehyde or 3∶1 methanol:acetic acid overnight at 4°C. Tissues were paraffin embedded and sectioned at 5 µm. Immunohistochemistry was performed as previously described [73]. Whole mounts of the retina were prepared for immunohistochemistry as described: Retinas were dissected and incubated for 15 min in cold buffer (50 mM NH_4_Cl, 0.02% sodium azide (NaN_3_ in PBS) twice and blocked overnight (0.01% Triton X100, 0.02% NaN_3_ in PBS). Retinas were incubated in primary antibody in blocking solution overnight at room temperature with gentle agitation. Retinas were then washed six times, 30 min each in blocking solution and incubated overnight in secondary antibody in blocking solution at room temperature. Retinas were rinsed six times, 30 min each at room temperature and mounted using SlowFade Light Antifade kit (Molecular Probes). The following primary antibodies were used at 1∶200 dilutions for immunohistochemistry, both sections and whole mounts: OPN1SW (goat polyclonal, SC-14363 Santa Cruz), Red/Green Opsin AB5405 (Millipore). The following secondary antibodies were used at 1∶400 dilutions for immunohistochemistry, both sections and whole mounts: Alexa Fluor 488 (Goat anti-rabbit, Invitrogen), Alexa Fluor 555 (rabbit anti-goat, Invitrogen).

### Western Blot Analysis

Western blot analysis was performed as previously described [18]. Briefly, retinas were homogenized in RIPA buffer (1 x TBS, 1% Igepal, 0.5% Na.Deoxycholate, 0.1% SDS, 0.04% Na.Azide, 1 mm PMSF), with 40 ug of protein utilized for each western blot. Primary antibodies used at 1∶500 dilutions unless otherwise indicated: *PRPF3* (rabbit polyclonal, Aviva); *THRAP3* (rabbit polyclonal, Lifespan); *MED1* (rabbit polyclonal, AbCam); *CERKL* (rabbit polyclonal, AbCam); *NRIP1* (*RIP140*) (1∶1000, rabbit polyclonal, AbCam); *NCOA2* (1∶1000, rabbit polyclonal, AbCam); *FOXC1* (1∶1000, goat polyclonal, AbCam); and *IRBP* (1∶200, goat polyclonal, Santa Cruz). The following secondary antibodies were used at 1∶10,000 dilutions: Alexa Fluor 488 (anti-rabbit, Invitrogen), Alexa Fluor 555 (anti-goat, Invitrogen), and Alexa Fluor 488 (anti-mouse, Molecular Probes). Samples were electrophoresed on 10% Tris-Bis NuPage gels (Invitrogen) according to manufacturer's recommendations. Proteins were transferred onto PVDF membranes and western blot analysis was performed using the Odyssey Infrared Imaging System (LiCor Technologies) according to manufacturer's recommendations. Blots were incubated in Odyssey blocking solution for 1 hour at room temperature. All primary antibodies were incubated in Odyssey blocking solution overnight at 4°C while secondary antibodies were incubated for 1 hour at room temperature. Images were visualized using Odyssey system and Odyssey v.1.2 software.

### Electroretinography

Electroretinogram analysis was performed on 7 mice of each strain (1–3 months). Mice were anesthetized with an intraperitoneal injection of a saline carrier (10 mg/g body weight) containing ketamine (1 mg/mL) and xylazine (0.4 mg/mL). Dark adapted (scotopic) electroretinogram recordings were performed using the UTAS E4000 system (LKC Technologies INC, Gaithersburg, MD) as described previously [18]. Mice were dark adapted for at least six hours and then anesthetized prior to recording. Signal processing was performed using EM for Windows v7.1.2. Signals were sampled every 0.8 ms over a response window of 200 ms. For each stimulus condition, responses were computer averaged with up to 50 records for the weakest signals.

### Pathway Generation and Analysis

Data were analyzed using Ingenuity Pathway Analysis (Ingenuity Systems, www.ingenuity.com). Gene identifiers and statistically significant expression values were uploaded into Ingenuity. Default cutoffs were set to identify genes whose expression was significantly differentially regulated and overlaid onto a global molecular network developed from information contained in the Ingenuity Pathways Knowledge Base. Networks were algorithmically generated based on their connectivity. Genes or gene products in the networks are represented as nodes, and the biological relationship between two nodes is represented as an edge (line). All edges are supported by at least 1 reference from the literature, from a textbook, or from canonical information stored in the Ingenuity Pathways Knowledge Base. Nodes are displayed using various shapes that represent the functional class of the gene product.

## Supporting Information

Figure S1 **Cone Specific Gene Expression.** Expression levels for multiple genes specific in their expression to cone photoreceptor cells are shown, as well as their chromosomal locations within both human and mouse. Expression levels are shown for each strain, relative to C57BL6/J, at both E18.5 and P30.5.
(TIF)

Table S1 **Standardized Microarray Expression Data Averaged by Strain.** Standardized microarray expression data is shown for both E18.5 and P30.5 time points with expression values averaged by strain.
(XLS)

Table S2 **Genes Showing Greatest Variation within Strains.** Genes exhibiting the greatest fold changes for each strain, at both E18.5 and P30.5 time points are shown. Gene symbols, corresponding GeneIDs, and p-values for each fold change are shown.
(TIF)

Table S3 **Microarray Data for Probes Correlating to Top Scoring Gene Networks.** Microarray data is shown in fold change (relative to C57BL6/J) for each strain at both E18.5 and P30.5 time points for each of the three top scoring 3098 gene derived networks.
(XLS)

Table S4 **Microarray Data for Probes Correlating to 120 Gene Networks.** Microarray data is shown in fold change (relative to C57BL6/J) for each strain at both E18.5 and P30.5 time points for each of the three top scoring 120 gene derived networks.
(XLS)

Table S5 **qRT-PCR Primer Design.** Gene names, abbreviations, amplicon size, and forward/reverse primers are shown for each primer pair used in qRT-PCR.
(PDF)
