# Correction: Genetic Variations Strongly Influence Phenotypic Outcome in the Mouse Retina

**DOI:** 10.1371/annotation/f375292d-9fc7-4e3c-9dc7-af0aee76fa15

**Published:** 2011-10-11

**Authors:** Austin S. Jelcick, Yang Yuan, Barrett D. Leehy, Lakeisha C. Cox, Alexandra C. Silveira, Fang Qiu, Sarah Schenk, Andrew J. Sachs, Margaux A. Morrison, Arne M. Nystuen, Margaret M. DeAngelis, Neena B. Haider

The authors wish to add the following funding sources to their financial disclosure information: RO1 EY017653 and The Edward N. Della L. Thome Memorial Foundation, Bank of America N.A., Trustee. 

