# Supplementary figures and images for: Genetic Variations Strongly Influence Phenotypic Outcome in the Mouse Retina
Source: PLoS One. 2011 Jul 14;6(7):e21858. doi: 10.1371/journal.pone.0021858 (PMC3136482; doi:10.1371/journal.pone.0021858)

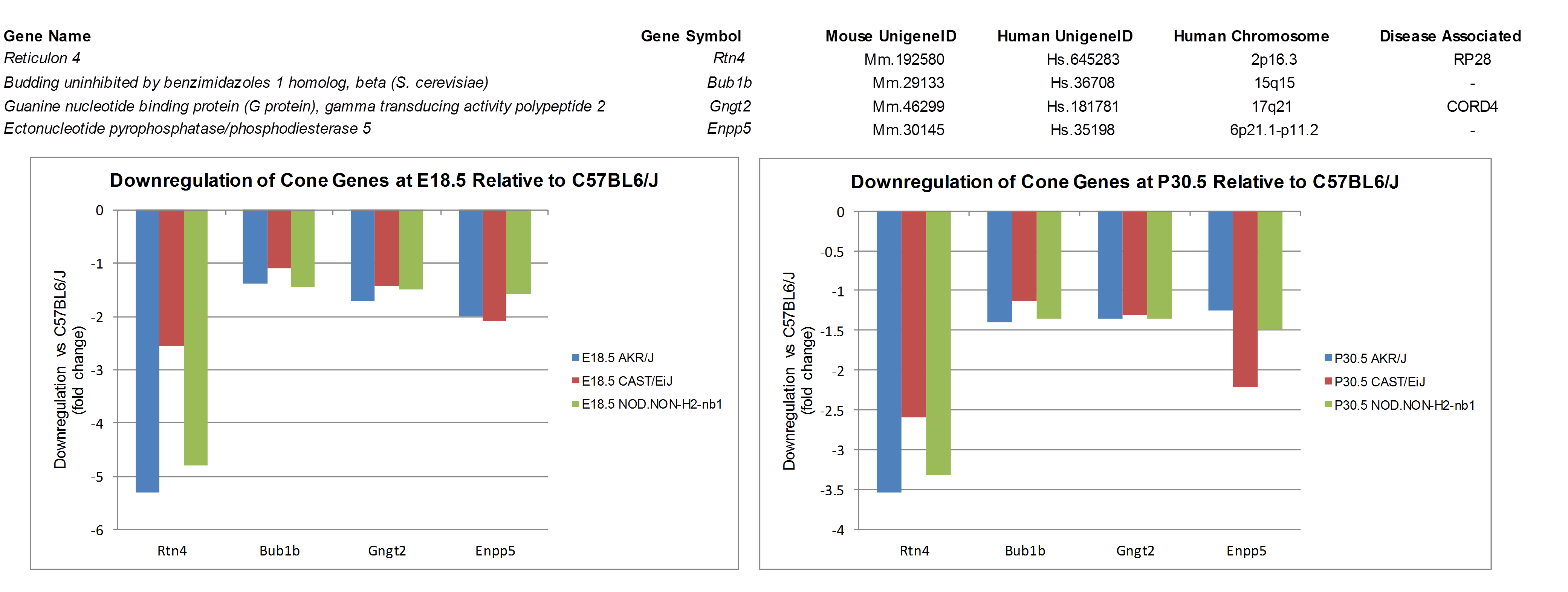

Supplement: Figure S1 — Cone Specific Gene Expression. Expression levels for multiple genes specific in their expression to cone photoreceptor cells are shown, as well as their chromosomal locations within both human and mouse. Expression levels are shown for each strain, relative to C57BL6/J, at both E18.5 and P30.5. (TIF) [file pone.0021858.s001.tif]

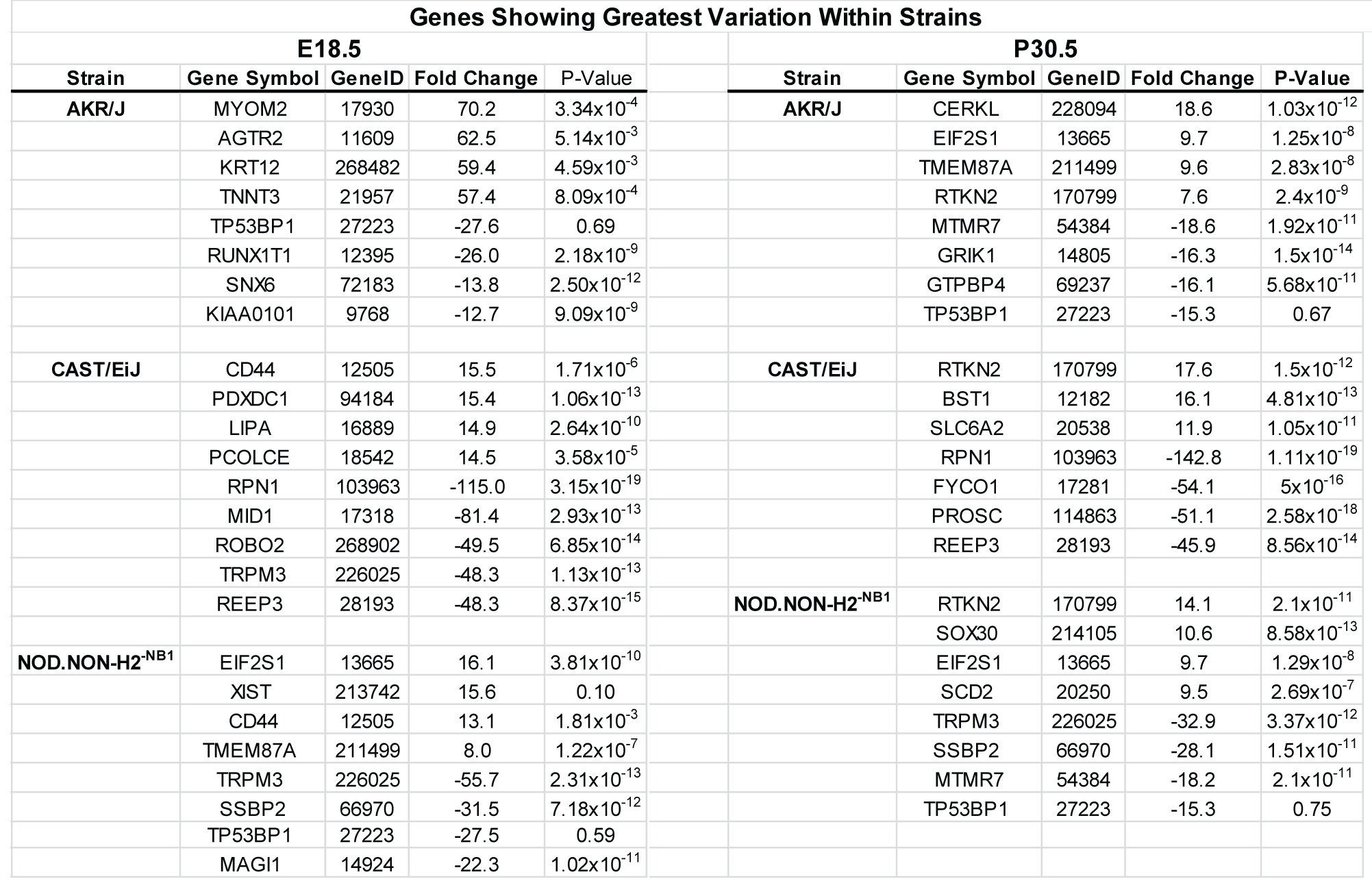

Supplement: Table S2 — Genes Showing Greatest Variation within Strains. Genes exhibiting the greatest fold changes for each strain, at both E18.5 and P30.5 time points are shown. Gene symbols, corresponding GeneIDs, and p-values for each fold change are shown. (TIF) [file pone.0021858.s003.tif]
