# Supplementary material for: Genetic Variations Strongly Influence Phenotypic Outcome in the Mouse Retina
Source: PLoS One. 2011 Jul 14;6(7):e21858. doi: 10.1371/journal.pone.0021858 (PMC3136482; doi:10.1371/journal.pone.0021858)
Supplement: Table S5 — qRT-PCR Primer Design. Gene names, abbreviations, amplicon size, and forward/reverse primers are shown for each primer pair used in qRT-PCR. (PDF) [file pone.0021858.s006.pdf]

qRT-PCR Primer Design

Gene Name	Gene Abbreviation	Amplicon Size	Forward Primer	Reverse Primer
	2900056m20		TCAGCCTGGTCTACAGAGTGAGTT	ATGCTCAAAGCAAACCTGGAA
	5330426p16	200bp	TGGTCTCACACAGTCTCATGAACA	CGGTTATCCCAATGCAGTTCTT
	A230097k15	200bp	GAACCATCTATCCAGTGGCCATA	AGCGGATTTTACCCTGTTTGC
	A330062j17	200bp	AGGCCCCTCCTGCTAGTTACA	ATGAGCTAACAGCCCTCAATTATCTT
	A930009a15	200bp	ATCCCGTTAGAGCAAACAACGT	TTCGTAGCAGGGAGATGACTCA
aldo-keto reductase family 1, member E1	akr1e1			
Rho, GDP dissociation inhibitor (GDI) beta	Arhgdib	200bp	TTCATGGTTGGCAGCTATGG	ACGGACAGACGCACTCATTCT
	b-actin	180bp	ATGCCTCCCCTACCAATCTTC	GGATAACGTCCAGGGAACCA
	bcdo2			
	bops	200bp	GAGGCCTTCTTGGGCTCTGTA	ATGAACCTGCTCCAGCCAAA
CAP, adenylate cyclase-associated protein 1 (yeast)	cap1			
carbonic anhydrase 8	car8	883bp	GCTGACCTGAGCTTCATTGAG	GTCTCTCTGGCTACTGAAAG
cholecystokinin B receptor	Cckbr	200bp	CGGTGGCCTATGGACTCATC	CACGTAGCAGCCATCACTGTCT
cyclin E2	CCNE2	200bp	ATGTCAAGACGCAGCCGTTT	GGGCTGATTCCTCCAGACAGT
carcinoembryonic antigen-related cell adhesion molecule 10	ceacam10			
cyclic nucleotide gated channel beta 3	Cngb3	200bp	ATACAGGGTCATATAATCGGAGGTCTT	TGTCCTGCTCCTCAGAGGTTCT
cellular retinoic acid binding protein I	crabp1			
	dmn			
DPH1 homolog (S. cerevisiae)	Dph1	201bp	TGTCATGATTGCCAACCCTAATATAC	TCCAAGTGCTCCAGGATCTTG
endothelin converting enzyme 1	Ece1		GAATACTGCAGGCGCCATTT	TGGCACCCGAACACTCTAGAC
endothelin 2	Edn2			
erythrocyte protein band 4.1-like 1	Epb4.1l1	193bp	AAGATGTCCTCACCAGCACGTA	TGCTGTAGTTTGGCTTCCTTGA
FGF receptor activating protein 1	frag1			
furry homolog (Drosophila)	FRY	200bp	TTTGTGGGATGTGTTTGTTTGG	TAGCCTAGCAACTTCAGAAAGGTACTTT
HRAS-like suppressor	HRASLS	200bp	CCAAATGCCTCTTAGGGATTCA	TTAGGGCTGCCCATTTTAAGG
importin 4	Ipo4		CAATGCCCGGGAAGCA	AACCACTCCGCGTACGTGTT
integrin beta 5	itgb5	200bp	CATCTCCACACACACTGTCGATT	GGTCCCTTAGGGCTCAATGG
inositol 1,4,5-triphosphate receptor 3	ITPR3	175bp	GGCCTGTGACACTCTACTTATGTGTATC	GAGGTTCAACACGATGATGATGA
mediator complex subunit 1	med1	195bp	GCTGGAAGCCCCAATAGTGA	CATTGGCATCCCCAATGTATTT
myocyte enhancer factor 2C	Mef2c	201bp	GACATTGTGGAGGCATTGAACA	GCTGGACACTGGGATGGTAACT
Musashi homolog 2 (Drosophila)	msi2	200bp	GAGAGATCCCACAACGAAACG	TTGCAGACAATCCTCCTACGAA
myosin, light polypeptide kinase	Mylk	201bp	CCAAACAGAAGCAGTTCCTAAGTCA	TAATTCAAAGTAATGCCCATCAGTATATCT
N-acetyltransferase 1 (arylamine N-acetyltransferase)	Nat1	200bp	GCTCTGACCAAAATGGGCTTT	GGCTGATCCTTCCCAGATGTT
neuron navigator 3	NAV3	200bp	AATTAGGTTGGAACTGACAAGATGCTA	CACTGTAAACTTTGAAAGAGGAAGAAAA
nuclear receptor subfamily 1, group D, member 1	nr1d1	191bp	CGGCTCAGCGTCATAATGAA	GTTGCCTTGCCGTAGACTGTT
nuclear receptor subfamily 2, group C, member 1	nr2c1			
nuclear receptor subfamily 2, group C, member 2	nr2c2	200bp	CTCACCTCAGCGCATTCAGA	GGTTGTCCGAGGTGGTGAATAT
nuclear protein 1	Nupr1	196bp	TACAGGACCTTGGAGAAATTAGGAGTT	TTTATTTCCCCCACAGTTCTTTTG
nyctalopin	nyx	200bp	CCACACACCCCCCCTTTTAT	GTGCCTATACTGAGGGATCAATTGT
olfactomedin 1	Olfm1	200bp	GGCGCCTATGACTACGATGAA	TCCATGTACCAGACCCGGTTA
	opnslw(bops)			
	Opsin1sw		AAGTTTTCCATCTACCTGGGG	CTCTTTGGTCGCCATGTTTGT
otopetrin 3	Otop3	70bp	CACTGCTGGGCCTTTTGG	CCACTCGCTTCGATTTGGAA
p21 (CDKN1A)-activated kinase 7	PAK7	200bp	GAGCCCCCCTATTTCAATGAG	GGTGGACCTGCCAATTTTAAGA
phosphodiesterase 8A	Pde8a	200bp	GGAATGGCAAGGAGTTTACCATA	TGCCTGCCTGGTTATCTGTTT
phosphoinositide-3-kinase adaptor protein 1	Pik3ap1	200bp	AGGCACACTGGAGAATGAGTACAC	CGATTTTAAAGGCCTGACACATAA
pituitary tumor-transforming gene 1	Pttg1	200bp	TGGGAAAAAGATCACCGAGAA	CCAGCCCTCTCTCTTCATTCA
retinol dehydrogenase 9	rdh9	200bp	TCAACGTCGCCAGTGTCTTG	CAGCATCTGGGTATTTGAGCATAA
receptor accessory protein 3	reep3	200bp	CTTGCATGGTTTCCCCTGTACT	CCTTGCCGTCCAAAATTCAC
	restat			
regulator of G-protein signaling 9	rgs9	200bp	ACGGGAGTCAGAATGCATAACC	CTGTCCGGCTTGAGGATGAG
sex comb on midleg homolog 1	scmh1	200bp	TAGCCAGCCTCTCTCCACAAG	TCTCTTTAAAAAAGGTCTGCACCAT
superkiller viralicidic activity 2-like 2 (S. cerevisiae)	skiv2l2	200bp	GCTCTTCCTGCAGATGAGGATTA	TCTCTTAAAGCCAATGCAATAGCA
solute carrier family 15 (H+/peptide transporter), member 2	slc15a2	200bp	TGAATATTTCCTTGGATGCCAAT	AAGACCCCGGTTGGTGATATTA
slit homolog 3 (Drosophila)	SLIT3	200bp	TTCCGCTTGCTCATGCAGTA	GAGCGATGTCCGATATCTGATTC
suppressor of cytokine signaling 3	Socs3	201bp	ATGCTGGCCAAAGAAATAACCA	TCTAAATGCTACTGCCTCTTCACAAC
SRY-box containing gene 30	sox30	200bp	AGAAAAAAAGCCTAGAATGCTTTGC	TCCAAGATAACGTAATGCTATCACATG
spermatogenesis associated 5-like 1	spata5l1		TGGGTCTGACACAGCCAAAG	TCATGACACTCCGACTAAGAATTTCT
spermatogenesis associated 9	spata9	200bp	TTGCCAGAGAAGCCAGACAA	TGGTCCTCTGGTATGTCTGATGA
single-stranded DNA binding protein 2	Ssbp2	200bp	ACAACCAGACCACCTTTTAGTTCCT	TCAATGCACGTATGCGTATATGTG
six transmembrane epithelial antigen of prostate 2	steap2	197bp	CTATCTTTCCCACCATTGTCTGTTT	CCACACCCAGCTTGTTGGTA
serine/threonine kinase 17b (apoptosis-inducing)	STK17B	200bp	GCAGTGGGACTTTGGAAGCTT	GCAAGGAGTCATCGAATCGAA
thyroid hormone receptor associated protein 3	thrap3	200bp	CTTGCACGACCTAGTGGTTTATTG	GAGGATCCAAAGTGATGCTCTTTAA
	Tyro3	200bp	TGTCGAAGGTGTGCCATTCTT	ACTCTGTGCGCTGGGTCACT
zinc finger, DHHC domain containing 14	Zdhhc14	65bp	GAGGACTGCGGACCAACATC	TTGGAGAGCCCAGCTGTCAT
ATP-binding cassette, sub-family A (ABC1), member 1	ABCA1	171bp	TTGGAGATGGTTATACAATAGTTGTACGA	TGAATATCCTGGCTAGAGATGACAAG
ATP-binding cassette, sub-family A (ABC1), member 9	ABCA9	200bp	CCACATGAAGTGGAACAAGAGGTA	CATATGCGATGCCTCGAGAGT
ATP-binding cassette, sub-family C (CFTR/MRP), member 4	ABCC4	200bp	CATCTGCGCCATCTTTGTAATC	TCTCTAGGTCCGTGTACTCAATCACT
ATP-binding cassette, sub-family C (CFTR/MRP), member 9	Abcc9	152bp	CAGATTCAAGCAACGCATGCT	AAGAGCCACTAATGGATGCAATG
expressed sequence AI662270	AI662270	200bp	TCCTGAGGACTTCTTGCTGTTG	GGTGGTGGTTGAATCAGTAATCTG
aquaporin 1	Aqp1	200bp	CTTTGGTTTGAGCATCGCTACTC	TGTCGACTAGGGAGGAGGTGAT
Rho, GDP dissociation inhibitor (GDI) beta	Arhgdib	200bp	TTCATGGTTGGCAGCTATGG	ACGGACAGACGCACTCATTCT
bicaudal C homolog 1 (Drosophila)	bicc1			
bone morphogenetic protein 4	BMP4	200bp	AGCCTTTCCAGCAAGTTTGTTC	CCTGAATCTCGGCGACTTTTT
C1q and tumor necrosis factor related protein 5 /// membr	C1QTNF5	200bp	CACCTCCTGCCACATTAAACAC	ACACTCGGTAATACCGTCACATACA
calmodulin-like 4	CALML4	200bp	TCTCCACCTTCCTGACCATCA	TTCAATGCCTGCTTCCTTGAA
cyclin D2	CCND2	201bp	AAAACCACCCAACCACATGACT	GCCCAAATTCACCAAACATGA
cyclin D3	Ccnd3	201bp	CCAACCTTCTCAGTTGCCAAA	AGCAGCAAGCAAAGCAACAGT
CD151 antigen	cd151			
CD44 antigen	cd44			
chloride intracellular channel 6	Clic6	200bp	TGGAATGGCATTTGTGTAAGAAAG	CAGAGCAACAACATCCTTAAAGCA
collagen, type XIV, alpha 1	Col14a1	200bp	GTGAAGACAGACAGTTTTCGAGTGA	CTTCCGTCATCAAGCACAGCTA
cellular retinoic acid binding protein I	crabp1			
Crx opposite strand transcript 1	crxos1			
cathepsin H	Ctsh	200bp	GAATTGCTCAGCCACCAAAAG	GCTCAGCCAAAGACAGCATTT
cytochrome b-245, alpha polypeptide	CYBA	200bp	AGGTTGGAGGCACCATCAAG	GACAACTCCACAGAAACTCAAGCA
EGF-like domain 7	Egfl7	200bp	AGAGCATGGGCTACAAGATCCT	CTGAACTCTGGCTACAAGGGATCTA
EH-domain containing 4	EHD4	200bp	CTGATCGAGGCTGTGGACAA	TCTTTAGCCACGACCCACTCTT
ELK3, member of ETS oncogene family	Elk3	200bp	CTCTATAAAAGTCTGTTTCGCATTCAGT	AGCTGGTTACATCGTGTATGCAA
ELOVL family member 7, elongation of long chain fatty acid	ELOVL7	200bp	ATGGGACCAGCCTACCAGAA	CGGTACCAAAAGTGGAGAAAGAG
forkhead box C1	Foxc1			
forkhead box P2	Foxp2	201bp	TAAACCCCAAAACTATGATAAGACGTT	TAGCAGCCACCTTCTGTCAAAAC
frizzled homolog 6 (Drosophila)	FZD6	200bp	CCTGATTGGACTGTCCCTTTCTT	AAGGAGCACCACACAGTGAGTTC
growth hormone receptor	Ghr	201bp	AGCTGATCTCTTGTGCCTTGATC	TAAAAGTCAATGTTTGCCAGTGATG
guanine nucleotide binding protein (G protein), gamma 11	GNG11	200bp	AACTGAAGATGGAGGTTGAGCAA	TCCCTCCCCCAGAGTTACTTATG
guanine nucleotide binding protein (G protein), gamma 2	gng2			
G protein-coupled receptor 116	GPR116	200bp	CTTGGTGTGCTGTCTGTATTGCT	CACGGGCATAAATTCAGAATCTAA
G protein-coupled receptor 177	GPR177	200bp	AGCTCAGTGAAAAGTGGATACCTAGTG	CCACCTGCTACAGATGCTGCTAT
gelsolin	Gsn	201bp	TACATCGAGACAGATCCAGCAAAT	AAGGCACTGATTGGTGACATTG
InaD-like (Drosophila)	INADL	198bp	GGTACACCTGACCCTTGTTCGA	GCTTGTACAGTGTTGTTTTTTAGATTATCC
jagged 1	JAG1	200bp	GAAGTTGGAAGCATCCCAATCTT	GCAGTGTACTCTACGGCAAATCTAAA
	jarid1d	201bp	GCCAGGATCTGACGACTTTCTAC	ATTCGAGGAGTAAATCTGAAGTTATCAA
Kruppel-like factor 4 (gut)	klf4	200bp	GGTCAAGTTCCCAGCAAGTCA	CGGGCATGTTCAAGTTGGAT
keratin 8	KRT8	201bp	AAGGCTGTGGTTGTGAAGAAGATT	GCCAGAGGATTAGGGCTGATC
LIM domain binding 3	LDB3	200bp	GTAATTCAGTGGTACAGAATTGTCCTAGAA	GTATTCCTCCTATGCACTAGTAAAGATGTG
LIM domain containing preferred translocation partner in li	LPP	200bp	CCACACCCTCAGAGCTGCTT	GTCACAGGCTTAGCCAGACGAT
mannosidase 1, alpha	MAN1A	200bp	GTGACAGCCTCTTAGCAGGTCTATC	TTAGAGCAAAAGCAGAGAACGAAA
mbt domain containing 1	mbtd1	200bp	ACAGGACGGACATTTCGATACAC	CTGCTGCTTCTGAGCCATCA
myocyte enhancer factor 2C	Mef2c	201bp	GACATTGTGGAGGCATTGAACA	GCTGGACACTGGGATGGTAACT
microphthalmia-associated transcription factor	MITF	200bp	CAGACATGCGGTGGAACAAG	GGTGGATGGGATAAGGGAAAGT
melan-A	MLANA	200bp	TGTGCTGAGTCCACACAATGC	ATGACGCCTTTTGTCCATCAA
Musashi homolog 2 (Drosophila)	msi2	200bp	GAGAGATCCCACAACGAAACG	TTGCAGACAATCCTCCTACGAA
nuclear factor of kappa light polypeptide gene enhancer in	nfkbia	200bp	CTGACCTGGTTTCGCTCTTGT	TGTGAATTCTGACTCCGTGTCAT
	NIBAN	201bp	TGAAGCCCAAGCCTTTTTAGAA	AGTCCAAACCAGGCCCTCTT
nischarin	nisch	201bp	TCACCTGTCGCCTTAAGTATCTTAAG	AGAATCTAACACTCATTGTGGCTAAAGT
nephronectin	NPNT	200bp	CTGTGTGTATATCCCCAAAGTCATG	TAGGTCTTGTTGTTGGCTTGGA
nuclear receptor subfamily 1, group D, member 1	nr1d1	191bp	CGGCTCAGCGTCATAATGAA	GTTGCCTTGCCGTAGACTGTT
nuclear receptor subfamily 2, group C, member 1	nr2c1			
nuclear receptor subfamily 2, group E, member 1	nr2e1	200bp	CGCGTGGAGAGAACTGTTTGT	AGGCAAATTCAGTGGCGTCTA
nuclear receptor interacting protein 1	nrip1	200bp	TCCGAGAACTGTGTGCGAGAT	TTTACCATTCCCGGGTATGAAA
nuclear receptor-binding SET-domain protein 1	nsd1	196bp	GGAACTGAAGGAGCTCTCGTACA	TAACCGCTGCTCCTTTGTCTTAC
protocadherin 15	pcdh15	200bp	TACAGAGATTTTGGATCGCTACGTT	GTTTGCCGTCCAGGAACTTAAA
PHD finger protein 15	PHF15	196bp	GAGTTAAGGGCCTCAGGTTTGTT	CCAAAGTCTAACCTGCTGCCATA
paired related homeobox 1	PRRX1	200bp	TGCTCTCGGATACCTACCATCA	CAAGCAGGTTACCGAGAAGTGA
	Pxx2	200bp	CCACGATGAGCCCAGATTATCT	GGACCCAATGCACAGTCAGTT
RAB38, member of RAS oncogene family	Rab38	200bp	CACCCCATCAGCACAGTGTTTA	GCAAGACAAATCCCCACACTTAG
retinoic acid induced 14	rai14	200bp	CTTTTGCCCGTTGAACTTGTAAA	AAGCTGCAAGCAAGTTGAACCT
retinoic acid receptor, beta	rarb	200bp	TTTTCCGCAGAAGTATTCAGAAGA	GCTCTCTGTGCATTCCTGCTT
retinoic acid receptor, gamma	rarg	197bp	GGCTTCTTCAGACGCAGCAT	GGCGAGCCCTCCTCTTTTAC
retinoic acid receptor responder (tazarotene induced) 2	Rarres2	200bp	TTAAAATGGACCCCAAGGGTAA	AGGTGAAGTCCTGTCTAGGGCTTA
RAS, guanyl releasing protein 3	RASGRP3	200bp	CTCCTCTGCATGTATCGGAATG	GATATGTCCATGAGGCTGATGTG
retinoblastoma-like 1 (p107)	rbl1	200bp	GAGGAGATTGGAACACCTCGAA	ACACTTTGAGTGGCTGAAGCTACA
retinol binding protein 3, interstitial	rbp3	200bp	CGGGACAGCCGTTATCTGTAA	CTACGGGCCACCATTGTCAT
receptor accessory protein 3	reep3			
retinol saturase (all trans retinol 13,14 reductase)	retsat	200bp	TCTTCATTGCCTTCCCATCAA	CAGTTTCATGATCACCGACATAGAG
ras homolog gene family, member D	Rhod	200bp	CAAGGTGCTGGTGAACAACCT	TAATCCGCCGCCAAAAGTTAT
ras homolog gene family, member J	RHOJ	200bp	ATATGGAATGCAGCAGGAAACC	CAGCCCGTGAGAATCTCACTCT
ring finger protein 128	RNF128	200bp	TCAAAGAGGCATCCAAGTCACA	CTGCCTTTAACTGCCTCTGCTT
RAR-related orphan receptor beta	rorb	200bp	CATGGAGCTGTGTCAGAACGA	ACAGATTCTTCGCAAAGTCAAATG
retinitis pigmentosa GTPase regulator interacting protein 1	rpgrip1	200bp	AGATGTCGCGTATGGCACTCT	AGGAATAAGTGCAGAAAGTGGTAGGT
	S10096			
sema domain, immunoglobulin domain (Ig), short basic do	sema3b	201bp	TGCCAGGAAGACAAATGAGTTATG	ACTCTGGAACCCCACGTCTCT
sema domain, immunoglobulin domain (Ig), short basic do	SEMA3C	200bp	CGTGCAGAAGGTCGTAGTCCTT	CACAGGCTGTGCCGTAGATATG
secreted frizzled-related protein 1	sfrp1	200bp	TTTGAACTGGCCACCTTTGATAG	ACCTTTGCTCATTTCTTCCACTCT
sine oculis-related homeobox 4 homolog (Drosophila)	Six4	201bp	TGGCCTTGAACTCACAGAGATTC	TAAATACGACAGGCAACAGTCATGT
Six6 opposite strand transcript 1	six6os1	200bp	AATTGTTACTGCCATGTGAGTCTCA	AGCTGGAATATGTGAAGATTTTGAATTAC
solute carrier family 38, member 4	SLC38A4	200bp	GCCATTAATAGAACATCGTGTGGTTA	TACTTGCATTTCAGACTTGGCTTTT
sclerostin domain containing 1	SOSTDC1	198bp	AAGCACCTGATGCTGCATTCT	AAACTAACCCCTGGCTGTCAAA
SRY-box containing gene 17	SOX17	200bp	TAGCTCAGCGGTCTACTATTGCA	CGTGTAGCCCCTCAACTGTTC
T-box 15	TBX15	201bp	TGCCTACAGGGATGGAGCAT	TGATCCACCCACAGAGATTCTG
T-box18	TBX18	200bp	CTAAGTGGATGGTGGCAGGAA	GACATGCACACGTGGTTGGT
transforming growth factor, beta induced	TGFBI	200bp	CGGTGTGGTCTATGCCATCA	CTCGGTCTTCCTGCTAATGCTT
transforming growth factor, beta receptor II	TGFBR2	200bp	ATGACCCTCTACAGTAGGGTGCTTT	TTTGATTTTTGGTAGCCCGTAAA
transforming growth factor, beta receptor III	TGFBR3	186bp	AGCTTTGTGTTCAAGTCCGTGTT	GGGCTTGGTGAATGTCTTCTTATT
tumor necrosis factor, alpha-induced protein 2	TNFAIP2	200bp	GAATGGCTCCACTGCAACCT	TGTTCCGGATGCTCCTGACT
tumor necrosis factor receptor superfamily, member 23	Tnfrsf23	200bp	TGTCCCTCAGGTACATTTGTCAA	CCTATTTGGCACTCGCATTTC
tumor necrosis factor (ligand) superfamily, member 12 ///	TNFSF12		TCCTTGGTCCAGTCCTGTCTCT	GTGCCTGGTGCACAAAGTCA
transformation related protein 53 binding protein 1	trp53bp1	200bp	TCATCTCTTACTGTTGAGTGTTCCAA	TCAGTATCGTCAATCTGTGTGTTCTC
transient receptor potential cation channel, subfamily M, n	TRPM1	201bp	ACATCTTTGGTGTCAACAAATATCTTG	AGATCATCCAGTATGGCATGTAGAAG
twist homolog 1 (Drosophila)	TWIST1	200bp	CCCACACCTCTGCATTCTGAT	AAAGGTGTTAAAATTCCTCTGATTGTTAC
uroplakin 3B	UPK3B	200bp	CTAGAGGAGGTGGCCAGTTCA	GCCTACCGGGTTGTGTGTTT
vestigial like 3 (Drosophila)	Vgll3	200bp	GAATGTTCTTCTCTGCCCCTCTT	AACCGACACAGTGTCAACATCTG
Wnt inhibitory factor 1	WIF1	200bp	TCTGAAGGCAACACCATCCTTA	ACACACAGACCACCGTTCATACA
WNT1 inducible signaling pathway protein 1	Wisp1	196bp	AAGTGATTTGCTCAAGTTTTTAACCA	ATCAGTCTCTAATCACCCTCGACAT
wingless-related MMTV integration site 5A	WNT5A	200bp	CTGCAGAATTCGTGGTGTGAAT	ACGGCGGTAATTAGGGCTTT
Yip1 domain family, member 4	yipf4	200bp	CCTCGGTGTAAGCGGAGATTT	TAAAACCCAGCGATGGCATT
zinc finger, CCHC domain containing 5	ZCCHC5	200bp	GGCCTGATAGTTGCGAGAACA	GAGAAGAAGCTGCCAACAAAGC
